# “*The pandemic made us stop and think about who we are and what we want:*” Using intersectionality to understand migrant and refugee women’s experiences of gender-based violence during COVID-19

**DOI:** 10.1186/s12889-022-13866-7

**Published:** 2022-08-01

**Authors:** Alli Gillespie, Ilana Seff, Camilla Caron, Maria Margherita Maglietti, Dorcas Erskine, Catherine Poulton, Lindsay Stark

**Affiliations:** 1 Brown School at Washington University in St. Louis, One Brookings Drive, Campus Box 1196, St. Louis, MO 63130 USA; 2 UNICEF, Rome, Italy; 3 UNICEF, Geneva, Switzerland; 4 UNICEF, New York, USA

**Keywords:** Intersectionality, Migrants, Refugees, Gender-based violence, COVID-19, Italy, Qualitative research

## Abstract

**Background:**

Migrant and refugee women have faced a myriad of challenges during COVID-19, which are often exacerbated by the interaction between this population’s diverse identities and established systems in the local context. This qualitative study uses the lens of intersectionality to understand migrant and refugee women’s experiences of gender-based violence and access to and quality of support services in Italy during the first year of COVID-19.

**Methods:**

Data were gathered from 51 key informant interviews and eight focus group discussions of 31 participants. Key informants included service providers across sectors, including gender-based violence and anti-violence organizations, government and law, health, psychology, social work, and anti-trafficking administration. Focus group participants were migrant and refugee women aged 18–65 from the following countries of origin: Bangladesh, Cameroon, Colombia, El Salvador, Gambia, Ghana, Honduras, Libya, Nigeria, Pakistan, Peru, Senegal, and Syria. Interviews were audio-recorded, transcribed and coded using a collaborative process with partners from UNICEF. Transcripts were then evaluated for arising themes using three methods of intersectionality analysis.

**Results:**

Data analysis revealed how COVID-19 converged with sexism, racism, and xenophobia in Italy, leading to increased public and domestic violence against refugee and migrant women. Another prominent theme was the exacerbated vulnerability for refugee and migrant women in precarious socioeconomic situations, which prompted many service providers to recognize and address gaps in service offerings and coordination around basic needs. However, due to resource constraints and bias, providers did not systematically incorporate inclusive language and cultural mediation into remote and online services, creating a heightened barrier to access for non-Italian women despite their complex needs. As such, refugee and migrant women highlighted community-based solidarity and support as protective factors during lockdown periods.

**Conclusion:**

Findings emphasize how overlapping dominant sociocultural and socioeconomic systems impacted refugee and migrant women’s experiences of violence during COVID-19 in Italy, and how some support services were unprepared to respond to the complex needs of diverse, newcomer populations. We discuss how policymakers and practitioners might consider intersectionality in their preparedness and response planning for gender-based violence services during health emergencies moving forward.

## Background

As the COVID-19 pandemic continues into a third year, with arising variants threatening natural and vaccine-based immunity, economies fluctuating, climate change exacerbating, and new political conflicts unraveling, addressing the health and well-being needs of multiple-marginalized populations is increasingly vital. One population that experiences compounding challenges is refugee and migrant women living in a reception context. Refugee and migrant women are defined in this study to include self-identified women who have experienced forced displacement or migrated voluntarily and who represent a range of formal legal statuses as recognized by national and international governance systems. Refugee and migrant women are at risk for violence at every stage of their transition through contexts and while crossing borders. For example, they might experience sexual violence in their countries of origin wherein conflict, health emergencies, and other economic or sociopolitical factors compromise their safety and force them to flee; trafficking along migration routes where they are vulnerable to exploitation due to reduced resources; and structural violence in destination countries where barriers such as legal status or discrimination may prevent them from seeking support [1–3]. A cross-sectional study sampling 503 migrants and refugees in the reception context of Italy found that nearly half had experienced violence since their arrival, with 40% experiencing at least one episode in the past year [4]. While people of all genders experience violence, refugee and migrant women are at an increased risk for gender-based violence (GBV), defined as “harmful acts directed at an individual based on their gender” [5], because of their migrant status combined with their socially ascribed gender as women. This study focuses on women because of their disproportionate risk for GBV across contexts and inside and outside of the home due to prevailing gender inequality, including social and economic power imbalances [5].

It is well documented that health emergencies such as COVID-19 disproportionately impact both migrants and women as separate groups [6–8]. One study showed that women seeking GBV support services in Italy reported high levels of violence prior to the pandemic, and nearly a third of women cohabitating with a partner experienced intensified intimate partner violence during March and April 2020 [8]. Meanwhile, a systematic review found that migrants living in high-income countries were disproportionately represented in reported COVID-19 cases and deaths during 2020 due to factors such as living conditions and healthcare barriers [7]. While the exact prevalence of GBV victimization is difficult to determine for migrant populations, a review of 84 studies consistently found that refugee and migrant women have a higher prevalence than local populations in both transit and reception contexts [9, 10]. More evidence is needed to address the disproportionate impacts of pandemic measures on GBV experiences for refugee and migrant women and to ensure their access to care as the pandemic continues and other health emergencies arise. As such, this qualitative study uses the lens of intersectionality to understand how refugee and migrant women’s experiences of GBV and access to support services were impacted during the first year of the COVID-19 pandemic in Italy.

### Context

Italy was one of the first countries to experience a large wave of COVID-19 incidence in early March 2020, with about 74,000 confirmed cases as of March 25th [11]. On March 9, 2020, Italy implemented a full lockdown lasting two months wherein residents were not allowed to leave their homes except for essential tasks and jobs; after a sharp decline at the beginning of the lockdown, requests for GBV support received by the national hotline 1522 increased, peaking in April (+ 176.9%) and May (+ 182.2%) [12]—presumably as a result of more people being confined indoors with their abusers and an increase in GBV awareness campaign**s** [13, 14].

At a higher rate than any other country in Europe, Italy has seen a substantial increase in its migrant population during the past 20 years, even with its complex, decentralized reception system and certain sociopolitical groups’ antagonism toward newcomers [15]. Hostility toward foreign-born populations, such as xenophobia stereotyping, is pervasive in some forms of Italian media, and systematic negative portrayals of migrant women in particular have been shown to compromise their physical safety in both public and private spaces [16–18]. Even as the pandemic unfolded, migration to Italy continued to surge, with newcomers arriving at the highest rate since 2017 during just the first nine months of 2021. During this time, incoming migrants were required to isolate in quarantine boats and other facilities before entering the institutional reception system [19, 20].

The International Rescue Committee estimates that nearly half a million migrants living in Italy are undocumented, largely due to a lengthy and fragmented asylum process that has led to crowded and resource-scarce reception centers scattered throughout the country [21]. In 2020, Italy served as the reception context for over 40% of Europe’s migrant arrivals, many of whom were refugees and asylum seekers [21]. While conditions in reception centers presented challenges prior to the pandemic, the spread of the virus created new and exacerbated prior health vulnerabilities and access barriers for resident migrants across the country [22]. For example, a quantitative study found that migrants living in reception centers in northern Italy had a higher incidence of COVID-19 as compared to the Italian resident population [23]. Further, a qualitative study conducted at a reception center in Bologna found that reception centers overemphasized the biomedical features of COVID-19 in their prevention and response efforts and failed to consider social determinants and outcomes—worsening structural violence and disproportionately impacting migrants who were undocumented [24]. Additionally, these centers often lack privacy and may not provide safe housing accommodations for people vulnerable to GBV or for survivors who have experienced GBV before or during their migration [25].

### Conceptual framework

The concept of intersectionality emerged from grassroots social liberation movements in the United States and the Global South as one method of analyzing differences in individual and group experiences [26, 27]. Black legal scholar Kimberlé Crenshaw coined the term “intersectionality” in 1989 as a metaphor for the multiple, interlocking oppressions Black women in the U.S. experience as opposed to single-axis systems such as sexism, racism, and classism [28]. Expanding on the concept in a 1991 article, Crenshaw argued that intragroup differences matter when addressing GBV; she highlighted how lumping together “women” as a category erases “the experiences of women of color” who “are frequently the product of intersecting patterns of racism and sexism” [29]. Further, she pointed to immigrant status as a factor which may alter women’s experience of domestic violence and support seeking as they navigate cultural and language barriers, gendered and exclusionary government policies, and increased dependence on abusive partners for material resources and legal information.

Intersectionality has become a widespread lens to address social and legal problems since its inception, but there is a lack of consensus among researchers and practitioners about how it should be defined and applied, especially in terms of public health research [30, 31]. In response to the critique of intersectionality as a vague theoretical concept, Choo and Ferree (2010) offer three distinct lenses of intersectional analysis and argue that centering a singular lens is undesirable and inadequate for effectively addressing inequity.

The ‘group-centered model’, most in line with Crenshaw’s original definition, focuses on inclusion and aims to amplify the voices and needs of those living at the intersection of multiple oppressive systems. For example, the group-centered model might explore how “the needs of women of color often [remain] invisible *as women*” in feminist spaces and “*as blacks*” within the U.S. civil rights movement [32]. One limit of this application, however, is that solely giving voice to marginalized women by telling their stories and highlighting their unique experiences runs the risk of positioning them as “others” against a mainstream audience of “normal” people.

Meanwhile, the ‘process-centered’ lens highlights interactions at the institutional level and interrogates the role of unmarked categories of people who hold privilege and power. In other words, process-centered analyses reject the notion that nondominant groups are “others” to be compared against dominant groups as a baseline; instead, the roles of both non-dominant and dominant actors (for example, women of color and white women in feminist spaces) are foregrounded.

Finally, the ‘systems-centered’ approach focuses on the dynamic and complex macro-level structures that perpetuate amplified disparities for people who belong to multiple marginalized groups. This approach requires examining the symbolic boundaries attributed to race, gender, and nation within particular social contexts, analyzing sociopolitical and media landscapes, and linking structural factors to the inequitable feedback loops they produce within institutions. For example, this lens might explore how racism operates within a feminist organization by examining how its policies perpetuate discriminatory social norms and leadership structures.

This study speaks to all three approaches: the study is group-centered in that it centers the lived experiences of refugee and migrant women during COVID-19, process-centered in that it also examines the role and perspectives of institutional service providers who hold positions of power in relation to refugee and migrant women, and systems-centered in that it analyzes the socially-constructed context and its interlocking, macro-level factors in influencing the range of migrant women’s experiences and needs.

Prior literature has used intersectionality as a lens to consider GBV and the migrant experience during COVID-19 [33–37]. Many of these studies use a systems-centered intersectional lens to theorize the risk factors and potential impacts of COVID-19 for refugees, asylees, and other immigrant populations. When considering migrant women’s experiences of violence during the pandemic, for example, these studies highlight inequitable labor and employment opportunities, restricted access to services and support, social isolation, and lockdown policies that increase GBV risk for individuals and groups with multiple marginalized identity dimensions. However, none of the studies are situated in the Italian context, and few of them attempt to center the direct perspectives and lived experiences of migrant and refugee populations. Further, many utilize single intersectional approaches rather than employing “complex intersectionality,” which moves beyond the inclusion of groups with multiple marginalizations to analyze also “the relationships that affect them intersectionally” [32]. As such, this study fills a gap in the literature by applying Choo and Ferree (2010)’s multiple approaches to intersectionality into a single framework to qualitatively understand migrant and refugee women’s experiences in Italy during the COVID-19 pandemic, and especially their experiences of violence and access to and quality of GBV services.

## Methods

### Data collection

This qualitative study, undertaken by the Center on Violence and Injury Prevention at Washington University in St. Louis and UNICEF, relied primarily on key informant interviews (KIIs) with service providers and other key stakeholders and focus group discussions (FGDs) with migrant and refugee girls and women. The research team conducted 51 key informant interviews and eight focus group discussions bringing together 31 women (see Table 1. Participant Demographics), for a total of 82 study participants. Initial key informants were identified through existing networks and subsequent informants were contacted using snowball sampling. Key informants included providers who serve migrant and refugee women across sectors, including within GBV and anti-violence organizations, government and law, health, psychology, social work, and anti-trafficking administration. All FGDs and most KIIs took place in person, though several KIIs were held virtually due to health and other considerations. The service providers worked at the national level, as well as in Lazio, Lombardy, and Sicily. Focus group participants were migrant and refugee women aged 18–65 from the following countries of origin: Bangladesh, Cameroon, Colombia, El Salvador, Gambia, Ghana, Honduras, Libya, Nigeria, Pakistan, Peru, Senegal, and Syria. Data were collected during the first year of COVID-19, so more recent asylum-seeking populations, such as those displaced from the Ukraine later in the pandemic, are not included in the study. Focus groups were held in person at reception centers, safe spaces, shelters, and intercultural centers. Groups were purposefully kept small due to COVID-19 precautions. Each focus group included 3–4 participants and was facilitated in Italian. Five of the eight FGDs included a cultural mediator to aid in translation and understanding between the participants and the facilitator, and their addition was deemed not necessary for the other three based on participants’ Italian language levels. Cultural mediators interpret communication differences and promote shared understanding within groups by providing clarity around cultural beliefs, norms, expressions, values, and orientations to life – moving beyond mere linguistic translation [38]. See Table 2 for the study’s original research questions and sample questions from the semi-structured KII and FGD interview guides.

**Table 1:** Participant demographics

No. of FGDs	No. of Participants	Data Collection Sites	Average Age	Age Range	Countries of Origin Represented
8	31	Palermo, Rome, and Milano	32.7	18–65	Bangladesh, Cameroon, Colombia, El Salvador, Gambia, Ghana, Honduras, Libya, Nigeria, Pakistan, Peru, Senegal, and Syria
**KII Group**	**No. of Interviews**	**Data Collection Region**	**Professional Sectors Represented**		
1	14	Lombardia	Health, social work, GBV and anti-violence, cultural mediation, social service administration		
2	17	Lazio	Health, social work, GBV and anti-violence, cultural mediation, communications, social service administration		
3	6	National	Social work, GBV and anti-violence		
4	13	Sicily	Law, health, social work, GBV and anti-violence, cultural mediation, social service administration

**Table 2:** Original research and sample interview questions

**Original Research Questions**
• What has been the impact of measures to contain COVID-19 on the safety and well-being of migrant and refugee women within and beyond the household? • To what extent were/are migrant women aware of GBV-related services during COVID-19 measures? Did this knowledge vary throughout the pandemic? What primary channels were available and used by migrant and refugee women to access reliable and understandable information on available GBV support services during COVID-19 outbreak? • How were GBV-related services impacted by the COVID-19 outbreak in terms of availability, accessibility, acceptability and quality across the three regions? How were GBV services adapted to effectively respond to the needs of migrant and refugee women during the pandemic and prepare for similar future crises?
**Sample FGD Questions**
• What was it like for you and other women in the community to be stuck inside during the lockdown? • We have heard that sometimes in this community, women may experience violence either inside or outside the home. When I say violence, I am talking about things like a woman being hit, slapped or punched, being yelled at aggressively or in a purposefully hurtful way, or being forced to do something sexually against her will. Have you ever heard of this violence happening to women inside or outside the home (or reception center) in this community? • How did this violence change during the lockdown and over the course of the pandemic? • Who do women talk to when they experience violence? How did this differ during the lockdown and over the course of the pandemic? • What do you think would help ensure that women who need such services are able to access them? What would help ensure women can access these services, during the lockdown specifically?
**Sample KII Questions**
• What is daily life like for migrant and refugee women in your community during COVID right now? • Suppose I was a young refugee/migrant woman who arrived in the area where you live who needed gender-based violence services such as hotline support, counseling, access to a safe space or medical care today, what should I know? • What safety strategies are migrant and refugee women in your community utilizing to prevent or mitigate experiences of GBV experienced in Italy or the reception site? • Can you provide some examples of how COVID-19 impacted the availability and accessibility of your services? • Since these public health measures employed in Italy to control the community spread of COVID-19, have you noticed any change in the needs of migrant and refugee women and girls who access your services?

The research team sought informed consent from all participants, obtained permission to audio-record, and ensured COVID-19 safety protocols, such as social distancing and the use of face masks and hand sanitizer, during all in-person activities. Participants were provided an information sheet explaining the study aims, design, and contact information and were briefed on the potential benefits and risks of their engagement. Further, participants had the opportunity to ask questions before consenting to participate and were informed that they could withdraw consent at any time. Participation in the study was voluntary, though FGD participants were reimbursed for any travel costs incurred. The Health Media Lab Institutional Review Board approved the study design and all study procedures.

### Data analysis

All study data were translated into English and de-identified upon transcription. After reading the transcripts and undertaking an initial round of memoing, the Washington University research team and UNICEF engaged in a collaborative process to develop two codebooks [39]: 1) around the impact of COVID-19 measures on migrant and refugee women’s GBV risks and experiences, and 2) around migrant women’s access to GBV information and services and the effectiveness of GBV programming during COVID-19. Team members co-coded select transcripts until 85% of excerpts were coded in the same way to establish inter-rater reliability. Once confirmed, the team coded all remaining manuscripts using the Dedoose software.

An inductive read of the data revealed both common and different experiences for migrant and refugee women in Italy based on their multidimensional identities and migration background; thus, a more systematic analysis was undertaken to understand these experiences using an intersectional lens. Excerpts tagged with the following codes were exported and reviewed for arising themes: intersectionality, harder to reach group or community, innovative adaptations or new service, job loss or employment challenges, lack of coordination, linguistic and cultural barriers, mental and emotional distress, and women or community led solutions, among others. The coded focus group discussion transcripts and key informant interview transcripts were also carefully reviewed to better understand participants’ experiences of violence and access to services, as well as service providers’ perspectives around these issues. Transcript excerpts were organized in a data display [40] to track how factors aligned with the three approaches of intersectionality analysis influenced participants’ experiences [32]; several questions guided the process of filling in this analytic table: did participants describe their experiences of violence and their perception of inclusion (in services and in society) in terms of their dynamic identity dimensions or make connections to their migration and displacement? How did refugee and migrant women and those providing them services describe their interactions with each other, and how did barriers, strengths, and power dynamics shift within provider/survivor and community relationships as the pandemic unfolded? In what ways did the systemic landscape in Italy influence refugee and migrant women’s experiences of violence and access to services? How did institutional policies and interactions (or lack thereof) contribute to this landscape?

## Results

**Fig. 1:**
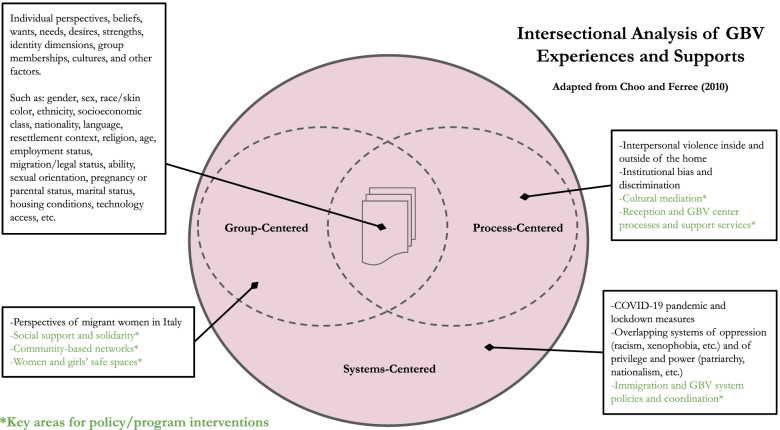
Intersectional analysis of GBV experiences and supports

Focus group participants and key informants described many challenges for migrant and refugee women in the Italian context, and there was a consensus that the COVID-19 pandemic, and especially the lockdown period, exacerbated these challenges. Several themes emerged, which highlight the intersectional nature of migrant and refugee women’s risks and experiences of violence and access to services within the Italian context based on their nationality, race, ethnicity, ability, age, religion, and socioeconomic status, as well as how these dimensions shaped their ability to overcome these challenges. These themes include 1) exacerbated risks and experiences of violence in public and in private spaces that occur at the intersection of racism, sexism, and xenophobia, 2) socioeconomic insecurity as a major risk factor for this population during the pandemic, 3) compromised access to GBV services in cases of ineffective incorporation of cultural mediators into remote services, and 4) the protective value of social support and community solidarity within and across borders. Table 3 provides an overview of these themes and their subthemes with an example quote for each, while Fig. 1 combines Choo and Ferree’s (2010) three methods of intersectional analysis to highlight key intersections emerging from the data.

**Table 3:** Results overview

Main Theme	Subthemes	Participant quote
**Violence exacerbated by heightened convergence of sexism, racism, and xenophobia**	Intersections in public	*“In my opinion, the pandemic has made things worse, because it has worsened how people see others, there is a widespread fear of ‘the other’.”* (FGD4—Senegal)
	Intersections in the home	*“For our husbands it is even worse. You have to keep quiet; other than that, they will beat you a lot. Five years ago the situation was bad, then in the last two years it got better, but now these episodes are starting again, only with the Blacks.”* (FGD3—Ghana)
**Socioeconomic insecurity as a prominent risk factor for violence and compromised service access**	Loss of employment	*“I think girls maybe are less safe because they cannot have a job anymore and cannot provide for themselves. No money, no cash, no security. They cannot afford houses and they live outside or in crowded places, this can be dangerous.”* (FGD1—Nigeria)
	Essential needs: barriers and supports	*“I was pregnant during the lockdown. My husband lost his job and we did not have good food. We ate pasta pasta pasta pasta, every day.”* (FGD1—Gambia)
	Fragmented coordination between service sectors	*“Even today, the reception system for migrants is divided into separate compartments. The legislation has been very fragmented. Extremely different rules have followed one another between 2018 and today. There have been enormous changes in terms of access to certain services, depending on the level of reception facility a migrant lives in. Migration procedures and regulations have become extremely confused and complex.”* (KII 49)
**Limited inclusion of translation and cultural mediation in online and remote services**	Institutional bias and discrimination	*“Not all women understand each other, there are no translators, and consequently in the end many troubles come out […] During the lockdown, therefore, there was a lot of tension, but the operators could not do much. We always try to talk to them about our problems, but I am not sure whether these problems are taken into consideration. Some social workers do not speak much English, so we cannot communicate much.”* (FGD8—Pakistan)
	Value of cultural mediation services	*“Women should be understood in public services. These offices need to hire foreign women speaking many languages, like cultural mediators!”* (FGD2—Bangladesh)
**Social support and community solidarity**	Protective factors for safety and well-being	*“I am currently a [job title removed] at the Women and Girls Safe Space, and even when we could not meet I always tried to talk to my students and fellow sisters over the phone, to keep up the work. The pandemic is being hard on us, but it’s nice to see many women finding the strength to do something for themselves and their future. Maybe one positive thing of the pandemic is that it made us stop and think about who we are and what we want.”* (FGD2—Ghana)

### Violence exacerbated by heightened convergence of sexism, racism, and xenophobia

Based on their multidimensional identities and circumstances, migrant and refugee women had varying experiences of violence and safety during the pandemic both in public and in private spaces. An arising theme at the systemic level was the convergence of sexism, racism, and xenophobia (see Systems-Centered section of Fig. 1), which was exacerbated during COVID-19, and which led to heightened discriminatory interactions and behaviors toward migrant and refugee women.

#### Intersections in public

Focus group participants highlighted that while migrant and refugee women generally felt subjected to more violence during COVID-19, their experiences of safety in public during the pandemic varied based on how their identities were perceived within the Italian context, as well as how they themselves perceived safety transnationally—in the Italian context as compared to their country of origin. A migrant woman from Pakistan explained how “*people have changed a lot. This disease is making people afraid.*” She went on to explain that “*Many people are dying, so there is a tendency of not trust*[ing] *anybody, of keeping others at distanc*e” (FGD8, Pakistan, 38). A participant in another focus group echoed this observation, emphasizing how multiple dimensions of her identity were ‘othered’ and how this othering impacted her:

*In my opinion, the pandemic has made things worse, because it has worsened how people see others, there is a widespread fear of ‘the other’. This summer I was working at the beach, making braids. People were afraid, few would let me get close to them. They didn't even want to talk to me. When you get close, people think you are sick, that you have COVID, or worse. First, they are afraid of the pandemic. Then they are also suspicious of another skin color. Plus, I don't speak the language well. Thus, fear and suspicion increased. Now we are used to bad things.* (FGD4, Senegal, 24)

As noted by this and a number of participants in our sample, discrimination was especially amplified for Black migrants and refugees at the intersection of anti-Black racism and xenophobia. In particular, Black women described increased harassment in the streets – both for them and their families. One woman described how people called her children “*monkeys*” and threw eggs at them. While she reported feeling safe at home during the lockdown, she shared “*we feel less safe now that we are out…now the situation is getting worse. People are stressed and unhappy, and they pour their problems on us*” (FGD3, Ghana, 38). Another woman from Ghana felt similarly, saying “*I do not know what is their problems, maybe they think they lost their job because of us, so they get angry with us, and insult us. They get angrier and angrier. Sometimes I really do not feel safe*” (FGD3, Ghana, 33). In such instances, respondents attributed some of the harassment to being scapegoated for losses felt by others in the pandemic; such interpersonal violence toward refugee and migrant women at the intersection of anti-Black racism, xenophobia, and health crisis aligns with the interaction or process-centered approach to intersectionality (see Process-Centered section of Fig. 1).

Migrant women from Bangladesh also described harassment in the streets. One woman pointed out how men in their communities were also targeted, though not to the same degree as women: “*Many foreigners got beaten here. We feel even less protected lately* […] *for women it is obviously worse, but violence also affects men. My brother-in-law was beaten only for a phone*” (FGD5, Bangladesh, 27). Another emphasized how she was met with mistrust regarding her adherence to mask-wearing because of her Islamic covering:

*Sometimes people got a bit unpolite because of my veil, because they thought I was not wearing a mask. It happened on the bus and in a supermarket that people stopped me because they thought I did not have a mask. When I told them I had it under the veil, they told me I had to put it out as well.* (FGD5, Bangladesh, 32)

Migrants who came to Italy as refugees or to seek asylum from violence in their home countries compared their experiences of violence, reflecting on how their identity was received in their new context. A woman from Pakistan said, “*When I was in my country I was in danger, but I have seen so much violence since I got here*” (FGD8, Pakistan, 24). She described how two women in her reception center “*often quarreled*” and “*were always looking for problems,*” with one even pointing a knife at the other. However, when she “*tried to do something to calm their tones,*” she was met with “*a bit of racism even among the women of the center,*” who “*told me to go back to my room and that those were not my business*.”

Women in one focus group who emigrated from Latin American countries described a different experience, with several of them reporting feeling safer in Italy than in their countries of origin. One woman proclaimed that she “*came to Italy precisely because I was not safe in Honduras*” (FGD6, Honduras, 65). She fled after people “*threatened to kill her entire family,*” so in Italy “*everything seemed calm to me, especially when I compare it to the reality we come from.*” Another woman from Honduras agreed, saying that “*even my children, when they arrived in Italy, were surprised that they could walk outside without any problems*” (FGD6, Honduras, 39). Even though she heard that certain areas of her Italian city were dangerous in the evening, she “*passed by there a couple of times and it wasn't like that for me.*” A woman from Colombia also added her perspective:

*I feel safe as a woman here. Of course, something can happen here as well when you walk in the streets, but people in Colombia go around armed. We are used to something else. Over there, you can commonly experience situations of violence and aggression in the street. I think women are much safer here.* (FGD6, Colombia, 32)

Refugee and migrant women described differential descriptions of safety and security, which were often constructed by their perceived inclusion in Italian public spaces based on comparative norms between contexts and the local prevalence of intersectional gender-based violence.

### Intersections in the home

Patterns of violence in public sometimes paralleled compromised safety in the home. Multiple participants noted that this was also true for migrant and refugee women in relationships with Italian men, due to multilayered power dynamics and their emergence in domestic interactions during the height of the initial COVID-19 lockdown (see Process-Centered section of Fig. 1). One woman said she “*trusted a person who convinced me to move to his house. He had promised to give me the Italian residence, marriage, children, everything*” (FGD4, Syria, 40). Instead, however, “*he told me not to work because I am a woman,*” then “*kicked me out of the house during the pandemic, in a bad situation.*” Another woman described a situation in which her sister sought help “*multiple times*” from “*the police, carabinieri *[Italian domestic military force]*, social workers*” after being “*threatened by her* [Italian] *husband*”—but she was not helped until her son was also threatened (FGD6, Honduras, 39). She perceived that the lack of intervention was connected to the multiple marginalized dimensions of her sister’s identity, including her nationality, migrant status, and skin color:

*My sister felt that there was an underestimation of the dangerousness of the situation she was experiencing. Even though she has regular documents, she had the feeling that these services have not promptly and adequately intervened because she is a migrant. If she was Italian, maybe they would have immediately taken this man and put him in jail. But they waited a long time before intervening, until it was my nephew who was attacked with a knife. Consequently, she doesn’t trust [the police and social workers] much, as she felt treated differently precisely because she is a migrant, with a different blood and a different skin color. My sister got deeply impacted by this delay, because it really took them a long time before they did something for her.*

Service providers also recognized this pattern and noted how abusive relationships with Italian men hindered migrant and refugee women’s access to support. The coordinator and operator of one anti-violence center and women’s shelter explained:

*Migrant women frequently suffer psychological blackmail from Italian men. "You are nobody, I am Italian, you are a poor migrant, etc.". They are constantly threatened and therefore they are more scared of accessing services, especially if they have children. If they have problems with their residence permit, they are less likely to ask for help* [from] *institutional services, due to the risk of being penalized on other levels.* (KII30)

In these cases, the intersections of power that Italian men held in terms of their legal status, gender, nationality, and skin color were important in their interactions and relationships with migrant and refugee women, as their intersectional power was sometimes leveraged to prevent their access to support services in a different way than for refugee and migrant women experiencing violence in public or within their cultural communities.

### Socioeconomic insecurity as a prominent risk factor for violence and compromised service access

Migrant and refugee women were described as being subjected to increased risk of violence and compromised access to support services based on their socioeconomic status within Italy (see intersection at the center of Fig. 1). Across groups, socioeconomic insecurity was reportedly worsened by the pandemic’s overall impact on employment sectors and its exacerbation of class inequities. This was especially true for migrant and refugee women, who often relied on informal labor or did not have the Italian language skills to participate in formal labor and thus relied on their spouses or other members of their family for livelihood support.

#### Loss of employment

While many women were at risk for violence inside the home during the pandemic, participants highlighted how “*the unemployed [refugee and migrant] women were more exposed to risks, because in the end they find themselves locked in a hole, with few future prospects*” (FGD6, Honduras, 39). Stress was amplified for some migrant and refugee women based on their legal status or their inability to access formal work opportunities. One woman explained how she had not been working since the COVID-19 outbreak began because of Italy’s lockdown measures (FGD7, Nigeria, 34). She shared, “*It is hard now to get any kind of job opportunities, especially in restaurants. I would like a legal job contract, but that is even more difficult!*” Some women also described being afraid to go to their informal jobs because of a lockdown policy that required people leaving their house to provide formal documentation stating their destination, which many did not have. An anti-violence center operator also reported on this increased vulnerability for many migrant women:

*The lockdown and the pandemic in general had a very strong impact on the working sphere and on the autonomy of women, especially migrants. Most of these women are engaged in care jobs, or they work in hotels as maids or housekeepers. These sectors have been particularly impacted by the pandemic and the COVID containment measures. Thus, their work independence has been reduced or canceled.* [...] *Most of them used to work illegally, so they were unable to access public subsidies. Therefore, they found themselves in a situation of serious vulnerability and further at risk.* (KII 25-26)

The lack of employment opportunities was reported to be especially prevalent for those who “*don't know the language well,*” with one woman describing how the situation was “*driving [her] into depression*” as she was “*falling behind comparing to the conditions [she was] living in [her] home country*” (FGD8, Pakistan, 38). She added, “*We have left a situation full of problems in Pakistan. But now we are worse off.*”

The lack of work opportunities made some migrant and refugee women more vulnerable by increasing their reliance on abusive partners. A focus group participant noted that when women had to stay at home “*during the lockdown,*” they “*had to go through their husband to do many things which they normally decide independently, so there were many discussions. Men argue a lot, and sometimes it doesn’t end up well,*” (FGD3, Ghana, 33) insinuating that women’s loss of autonomy via employment was a direct risk factor for domestic violence. Participants highlighted that migrant and refugee men were more likely to lose their employment during the pandemic compared to non-migrant men, and the stress of not being able to provide for their family—which may have cultural importance—sometimes was perceived to contribute to their perpetration of violence against their wives. A focus group participant explained one such situation:

*I know Senegalese men who used to earn their living by selling bags, but in this pandemic period they could not go out to do their jobs, they stayed at home, with their wives and children. [...] All that stress easily turns into violence. [...] They used to provide for their families, but since the outbreak of the pandemic they were no longer able to do their duty. Lack of money, especially for a proud person, can create a lot of problems and stress.* (FGD4, Senegal, 41)

Loss of employment was also a risk factor for violence outside of the home. One woman reflected on how younger refugee and migrant girls had an amplified risk for job loss during the pandemic, which impacted their ability to afford housing and thus made them vulnerable to violence on the streets or in crowded areas (FGD1, Nigeria, 21). Women whose economic security was compromised because they were “*leaving a violent situation*” had an “*increased risk of economic violence,*” a psychologist pointed out, adding that “*these are structural problems, but the pandemic has given the final blow*” (KII 5). In other words, effective support mechanisms for migrant and refugee women were limited before the pandemic and were further compromised by lockdown measures and the subsequent loss of livelihood opportunities. Another participant perceived that the economic impact on society as a whole compromised women’s safety in public, saying that “*to increase the level of women*[s’] *security outside, more jobs should be created. Social tension would then decrease, and we will have less episodes of violenc*e” (FGD5, Bangladesh, 36).

#### Essential needs: barriers and supports

Further, job loss and other changes in the economic system as a result of COVID-19 led to the loss of employment income and other sources of money during the pandemic. Many focus group participants highlighted compromised access to their essential needs during the pandemic, including access to food and groceries, safe housing, legal support, technology, resources (especially those in their language), education, healthcare, and space and privacy during the pandemic. One woman pointed out that the “*75 euro a month per person*” that the reception center provided was *“not enough!*” as her family still did “*not have money for diapers, for example*” (FGD7, Nigeria, 26). Another woman highlighted that “*The real problem during the pandemic was the collapse of the economy and the loss of job. Money. Cash. There was a scarcity of money, and a scarcity of food*” (FGD1, Nigeria, 21).

Some women described how the inability to meet their basic needs during the lockdown impacted their experiences of pregnancy and childbirth, and further decreased their access to health care. One woman who was pregnant described how her husband’s job loss limited her ability to acquire and consume nutritious foods; she also shared that she “*decided not to go to the hospital at the beginning [of the COVID-19 outbreak]*” because she was “*afraid that the hospital was full and that I would have not received a good treatment, so I stayed at home*” (FGD1, Gambia, 21). Pregnant migrant and refugee women also described missing appointments due to difficulties navigating online appointments or because of COVID-19 exposure or fear of exposure. As one woman shared:

*I went through a terrible time during my pregnancy. I was 38 weeks pregnant. A classmate of my son tested positive for COVID, but my son could not do a test for a while, and consequently I skipped some monitoring visits for this reason because I was stuck for some time at home.* (FGD5, Bangladesh, 32)

One of the younger focus group participants added that it was not just “*being pregnant during the pandemic*” that “*was very hard,*” but “*also giving birth;”* her experience as a young immigrant woman in a healthcare setting was compounded by the challenge of universal PPE requirements: “*they wouldn’t allow me to remove my mask* […] *It was very uncomfortable and I cried a lot*” (FGD1, Nigeria, 18).

Many service providers recognized migrant and refugee women’s increased vulnerability for violence or compromised service access due to socioeconomic precarity, and they made efforts to mitigate these risks by incorporating new service offerings during the pandemic. One informant pointed out that many migrant women “*did not have the right to access welfare subsidies*” offered to Italian citizens during the pandemic (KII 16); as such, organizations filled this gap in support by providing food distributions on site or via mobile units, grocery cards, health education and promotion, telephone support, legal and psychological support, and referrals to safe housing and shelters.

#### Fragmented coordination between service sectors

Participants, and especially service providers, spoke of how a lack of coordination between sectors was a systemic issue contributing to reduced access to services, especially for economically insecure migrant and refugee women and/or those without confirmed documentation of their migration status, putting these groups more at risk for GBV and other adverse outcomes (see Systems-Centered section of Fig. 1). A service provider illustrated this systemic inadequacy:

*The collaboration and communication between GBV services and reception structures are often not fluid. The Italian reception centers system is articulated, complex and heterogeneous, plagued by cuts in resources that have increased the precariousness and turnover of those who work in the reception facilities and transformed GBV services’ patient work of weaving relationships and networks in the territories into a Penelope canvas. Due to this poor collaboration, refugee and asylum seeker women do not always have access to information and knowledge of the territory.* (KII 16)

The lack of coordination directly impacted migrant and refugee women, with one asking, “*How can we feel safe here* [reception center]*?*” She explained: “*Operators of this place do not take care of us! They keep on telling us that there is nothing they can do, but there are many problems here, and we do not feel protected and assisted,*” and urged, “*We need to be put in contact with services, but this is not happening at the momen*t” (FGD7, Cameroon, 27).

Many service providers gave accounts of a decentralized and inhomogeneous system related to GBV emergency interventions, with “*each territory* […] *left free to organize the game as it wishes, and the central government does not even check and monitor these processes*” (KII 42). This key informant, who was the coordinator of an anti-violence center, also reported the role of politics in the perpetuation of inequitable outcomes for women during the pandemic:

*In the end, who pays for all of this? Women. Who found themselves in the middle of this chaotic and crazy system. The problem is that there isn’t a political will to change this situation. We have reported these challenges several times, but no action was taken, due to political parties’ games power.* (KII 42)

While women of many identities and backgrounds were likely impacted by incoherent GBV policies across territories, migrant and refugee women were further impacted by how the GBV system intertwined with a fragmented reception system. One service provider described how migration procedures and regulations had become increasingly complex and confusing in the years leading up to the pandemic, resulting in the restriction of refugee and migrant women’s access to services (KII 49).

Some migrant and refugee women, not knowing which services they had access to and which they did not, turned to the police as a first point of contact. A cultural mediator explained:

*Generally, the emersion of episodes of violence experienced by women, especially migrants, is difficult. During the pandemic, many thought that the services were closed, so in case of need they turned only to the police, because all the other services seemed to have disappeared.* (KII 41)

This was confirmed by participants in five out of eight focus groups in their responses to the question of who they contacted or who they would contact in the event of violence, with participants claiming: “*I did not know where to go, I did not know any service, the only one I knew was the police, so I called them*” (FGD4, Senegal, 41) and “*Even during the lockdown, the police responded. I do not really know if someone would have answered her or what would have happened. In any case, she should have called the police*” (FGD5, Bangladesh, 27). Although they were often migrant and refugee women’s primary form of emergency support, the police’s capacity to support survivors of violence inside the home was slowed down during the pandemic, sometimes increasing their exposure to violence. One woman shared how the police’s inaction caused her increased physical harm:

*The first time I called the police, they were not reactive, the second time as well. They told me "Madam what can I do? return to your home, we must all stay at home." I said "Arrest me, I can't stand it anymore." But they did not. They told me to go home, but that was not my home. I returned there, and again: Bang, Slash, Bang.* (FGD4, Syria, 40)

### Limited inclusion of translation and cultural mediation in online and remote services

One major theme among participants and providers was how institutions were frequently ineffective at incorporating inclusive language and cultural mediation into services, especially into remote and online services, creating a heightened barrier for non-Italian women during the pandemic, despite their complex needs (see Process-Centered section of Fig. 1). A key informant explained the role of cultural mediators as “*not as an interpreter, but as a person who represents the bearer of their culture, their messages of communication, of identity*” (KII 2). In some cases, there seemed to be a systemic feedback loop wherein some service providers claimed that migrant and refugee women did not seek GBV or support services due to their language and cultural barriers, which led to less funding or availability of interpreters and cultural mediators, consequently making it less likely for them to seek services.

The coordinator of one women’s organization noted “*sometimes their culture is precisely what is causing some barriers; for example, some have a cultural tendency not to discuss about personal problems outside the family*” (KII 40), while the coordinator of an anti-violence centre echoed that “*cultural beliefs and stereotypes can also hinder migrant women’s access to services*” (KII 38). Service providers highlighted how barriers related to language were heightened during the switch to online services, with one cultural mediator pointing out that “*many services are only by reservations, and many migrants do not know who to call and sometimes they cannot communicate over the phone”* because *“they are not familiar with the language and with technology*” (KII 41).

#### Institutional bias and discrimination

Due to budget and resource constraints, service components that benefitted migrant and refugee women were sometimes framed as add-ons rather than as holistic, inherently biasing institutional interactions and relationships (see Process-Centered section of Fig. 1). Some service providers recognized these gaps and advocated for adjustments to make holistic, quality services more accessible for refugee and migrant women. For example, multiple service providers reflected on persistent cultural biases and stereotypes within institutions. One key informant explained:

*…some GBV service operators have stereotyped and culturally insensitive perspectives which end up crushing migrant women in the role of victims, disregarding their strength and resilience and limiting the opportunities to rebuild their lives independently, express their needs, make their voices heard.* (KII 16)

Some service providers felt that a “*normalization of violence*” in migrant communities was what was hindering help-seeking and the self-awareness “*to get out of*” situations of violence; for example, one psychologist described what they perceived to be a “*cultural problem*” wherein migrant survivors “*talk about these episodes with resignation: ‘this has already happened to me. You know how it works in Libya, all women are raped, and all men are enslaved’*” (KII 23). While these service providers seemed to blame “*culture*” for migrant women’s attitudes toward help-seeking, a cultural mediator (who was also a migrant woman living it Italy) shared that they felt migrant women’s behaviors were more a result of a lack of education around their right to physical safety:

*In my opinion, many migrant women are not aware of what violence means, so even when it happens to them, they do not know how to recognize it and therefore they do not talk about it to anyone.* (FGD 5)

Although the anti-violence hotline offered multiple language options, many other institutions did not offer translated online support or outreach. For example, participants spoke about a GBV services awareness campaign launched during COVID-19 that was “*only in Italian,*” although one cultural mediator was encouraged that “*some messages also passed just through the images, so even women who did not understand Italian could have a sense of it*” (KII 18). Service providers felt that a lack of translation was less problematic when services were in-person because “*gestures can often help to ease the communication,*” (KII 18) and migrant women pointed out how this barrier worsened during COVID-19, with one participant sharing:

*Not all women understand each other, there are no translators, and consequently in the end many troubles come out. Many do not know or do not respect the rules. During the lockdown, therefore, there was a lot of tension, but the operators could not do much. We always try to talk to them about our problems, but I am not sure whether these problems are taken into consideration. Some social workers do not speak much English, so we cannot communicate much. Sometimes you need an intermediary to talk to them, and this is tiring and not that efficient.* (FGD 8, Pakistan, 24)

One key informant felt that “*social distancing has favored a cultural distancing on the part of the institutions*,” with more limited processes to support shared understanding across cultures during the pandemic resulting in “*increased institutional violence against migrant women*” (KII 42) (see Process-Centered section of Fig. 1).

#### Value of cultural mediation services

Across focus groups and key informant interviews, participants highlighted how services were sometimes inaccessible to migrant and refugee women from various countries and with different needs based on their identities, and spoke to how a lack of cultural mediation in services was exacerbated during COVID-19. Despite the limited inclusion in institutions and services, the value and importance of cultural mediators within GBV and other social services could not be overstated by study participants (see Process-Centered section of Fig. 1). Across FGDs, migrant and refugee women highlighted that the lack of cultural mediation in institutional interactions was a direct reason their peers chose not to access services:

*Many women will not access these services for cultural reasons. Maybe cultural mediators can do something about it.* (FGD3, Ghana, 33)

*Women should be understood in public services. These offices need to hire foreign women speaking many languages, like cultural mediators!* (FGD2, Bangladesh, 51)

Some service providers also recognized the vital nature of cultural mediation in services and described the actions they implemented to try and bolster cultural mediators’ presence and capacity within services. One key informant mentioned how their agency led “*substantial external and internal advocacy initiatives*” to “*welcome and support the specific needs of refugee and asylum seeker women,*” including taking steps to “*give a voice to cultural mediators, who are carriers of a feminist approach which is hugely different from ours. We have organized debates and discussions on this subject*” (KII 16). Another informant discussed how they engaged existing cultural mediators in making a small comic booklet on migrant and refugee women's rights in seven languages that could be downloaded online from their website (KII 38). This key informant also reported plans to “*distribute these booklets in pharmacies and offices of general practitioners; basically, in all the places women frequent, particularly those where there is a waiting time, when women can easily take something from the table and leaf through it.*” Some providers who recognized the importance of cultural mediators cited a lack of budget and resources as the reason they were not effectively incorporated into remote services during the pandemic.

### Social support and community solidarity

Some migrant and refugee women preferred to resolve issues of violence within their communities rather than involve public social services, emphasizing strong support networks as a protective factor within their communities (see Group-Centered section of Fig. 1). As one participant explained, “*We women of the Ghanaian community, when we have problem*[s] *with our husbands, we talk a lot between us, we share*” (FGD3, Ghana, 33). Another Ghanaian woman explained that “*many women do not go to these services, for a cultural reason. They try to solve the issue through other ways, internally*” (FGD3, Ghana, 38). She elaborated on the role of community elders in this process:

*We Africans, and especially we Ghanaians, if we have problems we talk to a fellow sister but not much to external people, such as professionals. When a woman from Ghana has a problem with her husband, the situation is managed within the “extended family.” The couple goes to elder people, to people who are considered wise in the community (and especially by the husband) and explains the problem. Some go* [to] *their parents in law. But many of us do not have parents here in Italy, so we use elderly as our parents. A woman goes to somebody her husband respect*[s]*, because this person can convince him to change behavior. Not the police or lawyers.*

In this example, support from elders was seen as an alternative to accessing services that may not be culturally resonant; however, sole reliance on this type of support may include other risks for women seeking to leave abusive partnerships or may be unavailable to migrants and refugees without strong community networks in Italy.

Focus group participants made intentional efforts to stay connected with their communities during the pandemic. They frequently spoke to their families and loved ones both within Italy and in their countries of origin, primarily via phone, Zoom, or the messaging app WhatsApp. One participant shared how she *“used to talk with some friends and family back in Bangladesh *via* phone already before the pandemic, but now it happens for friends and relatives here as well*” (FGD2, Bangladesh, 51). In another focus group, a woman explained that her “*mom communicates a lot with our family in Honduras over the phone*” so that “*she feels less the distance,*” and instead feels “*as if she was still in Latin America*” (FGD6, Honduras, 39). She added, “*these apps can help a lot.*” Finally, another FGD participant found her ability to communicate more with loved ones a silver lining during the lockdown period: “*I mainly talk to family and friends back home, and we always communicate *via* phone, so it did not change much for me. We all actually had more time to catch up, because we spent more time at home. So that was nice*” (FGD3, Nigeria, 25).

Not all migrant and refugee women had access to the tools needed for social and community support outside of their households. One woman pointed out that “*It is hard for some women to stay connected with their friends online because internet is expensive!*” (FGD5, Bangladesh, 27). In another focus group, a participant echoed this concern, saying that although “*new generations are good with technology,”* it was a major barrier that *“migrant girls here* […] *they do not always have the instruments to talk to friends. Some have connections only for couple of hours a day. This problem should be solved*” (FGD2, Ghana, 34).

Moreover, some participants described how their household family and spousal relationships were strengthened during the lockdown period. As one participant shared,

*The lockdown was not easy, but we were able to stay united as a family. With* [a] *normal lifestyle, the husband goes to work, the children go to school, and we do not spend a lot of time together. I was afraid for the developments of the situation outside, but inside my house I was feeling safe, finally spending quality time with my family - even if sometimes we did not know what to do!* (FGD2, Bangladesh, 51)

Another participant said that “*some of my friends were very happy* [to be] *at home, because they could spend more time with their boyfriends and husbands. Many women I know got pregnant during the lockdown!*” (FGD1, Nigeria, 19). Some participants appreciated their newfound quality time with spouses, with one woman sharing that “*before* [the pandemic] *it rarely happened, because he worked and he was always busy*” (FGD2, Ghana, 34). This participant also shared that she “*felt safe during the lockdown,*” and “*I had the affection of my husband and my kids, and they had mine, nothing is more important than this*.”

#### Protective factors for safety and well-being

Inclusion in strong social support networks and community solidarity were protective factors for migrant women during the pandemic in terms of their well-being and safety needs (see Group-Centered section of Fig. 1). Multiple key informants explained this link. As the coordinator of a reception center shared,

*Not being able to go out, many women have used smartphones to create social support networks and to share the difficulties they were facing. Of course, social media are not the same* [as] *social interactions in person, but this has partially compensated for the lack of moments of aggregation and helped to bring out some situations of vulnerability.* (KII 5)

One social worker mentioned a mutual aid network that mothers created (KII 36–37), while a cultural mediator discussed “*several episodes of great solidarity between migrant women*” she had been told about since the start of the pandemic (KII 18). For example, “*a group of Moroccan women collected and sent money to a woman who was suffering violence but did not know how to escape it,*” and the money allowed her to go to a bed and breakfast for a few days while finding a more long-term solution at a safe shelter. Another key informant who provided services as a psychologist “*noticed that people tried to cope with the pandemic as a group, as a community,*” as “*the community is a source of great resilience*” (KII 23).

Several participants reflected on the role of the women and girls’ safe spaces in facilitating their collective well-being during the pandemic. One shared that she was “*having problems with the COVID restrictions: the masks are painful, I do not like to stay away from people. We are not comfortable, we are not free,*” which was “*why I like coming here. Here we gather together, we do stuff as a group, we feel normal.”* (FGD2, Ghana 42). Another described how the safe spaces gave her a community, a job, and hope for her and other women’s future during the pandemic:

*Everything fell apart, in the whole world. Everything changed for us as well. We stopped going to work. You woke up every day in your house and you had to stay there, and then a day went by and another started, with the same routine. A routine of nothing. Everything changed totally in our lives. I was not going to work and I missed having some moments for myself and to socialize. This is why I decided to contribute to the opening of this Women and Girls Safe Space, to find a place where we could gather as women and do activities together, trying to find back some sense of community that got lost during the pandemic. I am currently a [job title removed] at the Women and Girls Safe Space, and even when we could not meet I always tried to talk to my students and fellow sisters over the phone, to keep up the work. The pandemic is being hard on us, but it’s nice to see many women finding the strength to do something for themselves and their future. Maybe one positive thing of the pandemic is that it made us stop and think about who we are and what we want.* (FGD2, Ghana, 34)

## Discussion

This study found that migrant and refugee women in Italy faced distinct risks for experiencing violence and compromised access to GBV services during the COVID-19 pandemic, and these risks were especially pronounced for migrant and refugee women who belonged to multiple marginalized groups in Italy based on their race, socioeconomic class status, religion, and other social dimensions of identity. Many participants named the socioeconomic impacts of COVID-19 as their primary protection concern; a lack of material resources impacted their ability to stay connected with their loved ones, feed their families, access healthcare and legal services, or afford stable housing in neighborhoods where they felt safe walking in public. Service institutions mitigated some GBV risk by offering new services such as food and housing support. However, many had challenges incorporating adequate translation and cultural mediation into online and remote services and struggled to coordinate with other actors across segmented immigration and GBV policy landscapes. Many migrant and refugee women emphasized solidarity and support within their family networks—both inside and outside of Italy—as integral to their resilience during the pandemic and valued the communities they were able to form in women and girls’ safe spaces. Figure 1 situates these findings within Choo and Ferree’s (2010) three combined lenses of intersectionality and offers key areas for program and policy interventions. This study adds to the literature by foregrounding migrant women’s lived experiences and exploring how these experiences were shaped by institutional services within broader systems. Further, this study offers insights into how practitioners and policymakers can better support migrant women—many of whom were refugees and asylum seekers fleeing humanitarian settings—in reception contexts during future health emergencies.

Participants in the study compared their experiences of well-being and violence in Italy to those in their countries of origin, situating their identity dimensions and group memberships in a certain temporal context and highlighting how their experiences of safety as women were tied to local perceptions of nation and belonging [41]. It is important to recognize that the convergence of racism, sexism, and xenophobia within the context of COVID-19 in Italy, specifically, contributed to migrant women’s experiences of violence and access to services. For example, Italy required documentation to leave the house during lockdown periods, inherently disadvantaging undocumented migrants or migrants working in informal sectors. Initial response efforts in Italy did little to address migrant populations’ social and legal precarities; instead, they focused on one-size-fits-all mandates to mitigate the biomedical aspects of COVID-19 and maintain national power structures [22]. The limited considerations for social determinants in COVID-19 response policy created a heightened risk of violence for migrant and refugee women, who described increased abuse in institutions and their homes during the lockdown period. Additionally, some faced harassment in Italian public spaces that in effect blamed them for COVID-19. These findings converge with historical trends of increased GBV in emergencies [42] and of European host societies scapegoating Black and brown immigrant populations during disease outbreaks and economic crises [43].

Findings from this study also resonate with literature exploring intersectional aspects of migrants’ COVID-19 experiences in non-European contexts. For example, Rieger et al.’s intersectional framework of GBV during COVID-19 in the U.S. theorized the protective factor of social support, the harmful response law enforcement may have toward minority women, and the impact of economic instability on both public and domestic GBV risk [36]. Further, a qualitative study of migrant workers in India found that when class inequities were exacerbated during the COVID-19 pandemic, social support among migrant women strengthened their hope for the future [37]. However, scant evidence has highlighted the role of culture in shaping migrants’ access to institutional support. Our results reveal the vital importance of interpretation and cultural mediation in GBV services for foreign-born women living in a reception context, especially in the transition to online and remote modalities during the initial lockdown period. This finding may be applicable across contexts and indicates the need for more widespread and explicit guidance on including cultural mediation services in emergency planning. Further, service providers in the study often perceived cultural mediators as an external resource used to reach migrant women with information about services and processes; less recognized were the ways in which providers might bolster their personal efforts to learn and understand the norms, values, and diverse needs of migrant populations and to tailor their service provision appropriately.

While scholars and the UN called upon policymakers and practitioners to center an intersectional approach in their COVID-19 response efforts within a few months of March 2020 [44–46], early dissemination of these strategies was lacking. For example, a guidance paper developed by the Gender-Based Violence AoR Helpdesk did not mention intersectionality or culture in its recommendations for how GBV practitioners might adapt case management service delivery models quickly and ethically during the current COVID-19 pandemic [47]. If similar guidance was lacking in the context of the present study, institutions may not have prioritized migrant and refugee populations in their early adaptations. Practitioners across contexts were left to fill in the gaps and develop innovative solutions for supporting survivors with differing needs and resources as the pandemic unfolded. For example, practitioners in high-income countries trained community members working at grocery markets and pharmacies to refer neighbors seeking help for GBV, while those in low-resource settings worked to ensure that safe spaces remained available during government lockdowns as an essential life-saving support [48]. These solutions resonate with this study’s findings and with past literature showcasing the effectiveness of women and girls’ safe spaces in facilitating social support and psychosocial well-being in humanitarian contexts [49].

However, innovative interventions during global public health emergencies cannot replace systemic change. More explicit policies and guidance are needed for GBV practitioners who serve migrant women, especially those living in high-income countries where interlocking systems of classism, racism, sexism, and xenophobia are insidious and pervasive. Ongoing implicit bias training across institutions and their hierarchies could help service providers to recognize their clients’ intersectional needs and strengths and to gain awareness of how they themselves perpetuate migrant women’s compromised access to service. These biases are systemic: Framing migrant and refugee women’s holistic needs as add-ons allows response systems to limit services such as cultural mediation when budgets are tighter. At the same time, some of the migrant and refugee women in the study preferred to handle experiences of violence within their communities rather than seeking formal services, with some indicating that this avenue allowed them to avoid potential cultural bias and discrimination.

In designing intersectional interventions during health emergencies, GBV policymakers and practitioners should engage migrant communities in planning and response processes and advocate for resources to be distributed equitably based on intersectional, community-defined needs, which may include hiring and training more cultural mediators or offering new services. Beyond community engagement and equitable budgeting, organizational leaders should change norms around who is considered the “standard” service recipient. The GBV Information Management System (GBVIMS) and the UNFPA GBV Area of Responsibility Community of Practice (GBV AoR CoP) are two promising resource-sharing networks that may help to facilitate practitioners’ and policymakers’ development of culturally-responsive GBV interventions across contexts [50, 51]. The GBVIMS is a standardized system for secure and ethical data collection and storage that providers can use to share internal data and coordinate more effectively with external agencies regarding GBV trends. Meanwhile, the GBV AoR CoP is an online forum wherein GBV specialists across contexts can build community and share information about protection needs and resources with the goal of improving GBV prevention and response.

### Strengths and limitations

Key strengths of this study include the ethnic and cultural diversity of focus group participants and the range of key informants’ service provision sectors. Additionally, Choo and Ferree’s (2010) three methods of intersectionality application provided a strong framework for thematic data analysis. The choice to combine the three methods into a singular framework allowed for a more holistic analysis of the ways in which refugee and migrant women were impacted by COVID-19 measures, and how their different identity dimensions presented unique challenges and strengths as they navigated complex systems and institutional processes. Further, using multiple approaches presented a richer understanding of how policymakers and organizational leaders can better support migrant and refugee women’s multifaceted needs. This method of intersectionality analysis can be replicated when conducting qualitative research with emerging and growing asylum-seeking populations, such as individuals and groups from Afghanistan, Ethiopia, Ukraine, Syria, and other emergency contexts not represented in the current study [52].

This study focused on refugee and migrant women based on their disproportionate risk of GBV; however, while an intersectional lens was applied, the use of a binary conception of gender to recruit participants and understand their experiences is a limitation. The results disrupt a single-axis story of “women’s challenges” during the pandemic, yet all focus group participants were those who identified, or were identified by the research team, as “women.” This study did not consider the perspectives of migrants and refugees who are gender non-conforming people, transgender people, and men who hold one or more marginalized dimensions of identity such as disabled men, LGBTQIA + men, non-white men, or poor young Black Muslim men. Notably, participants in the study highlighted that their communities were also impacted by violence against men due to xenophobia and socioeconomic instability. Lokot and Avakyan (2020) rightfully point out that gender is “often invoked as a lens through which to understand inequalities affecting [women and girls],” but may also “obscure how a number of intersecting oppressions further disadvantage certain people” [53]. Non-women who hold multiple-marginalized identities also deserve support in order to reduce violence victimization or perpetration and other adverse health and well-being outcomes such as illness, death, poverty, and family separation. Future qualitative studies exploring the intersectional impact of COVID-19 on GBV might consider expanding participant eligibility to gain a more comprehensive understanding of community needs and strengths within resettlement contexts.

## Conclusion

GBV service providers and institutions play a key role in supporting migrant and refugee women who have experienced public or private violence in resettlement contexts, yet the COVID-19 pandemic has exacerbated many systemic inequities that compromise their access to services and support. This study highlights the value of migrant and refugee women in Italy’s lived experiences in uncovering intersectional challenges and promotes the engagement of migrant communities in designing and implementing GBV support and advocacy strategies as the global community continues to address the COVID-19 pandemic and confronts other public health emergencies.

## Data Availability

The data that support the findings of this study are available from UNICEF Italy, but restrictions apply to the availability of these data, which were used under license for the current study, and so are not publicly available. Data are however available from the authors upon reasonable request and with permission of UNICEF Italy.

## Abbreviations

COVID-19: Coronavirus disease
FGD: Focus group discussion
GBV: Gender-based violence
GBVIMS: GBV Information Management System
KII: Key informant interview
LGBTQIA+: Lesbian, Gay, Bisexual, Transgender, Queer/Questioning, Intersex, Asexual, etc.
UN: United Nations
UNICEF: United Nations Children’s Fund
UNFPA GBV AoR CoP: United Nations Population Fund GBV Area of Responsibility Community of Practice
